# Green Extraction of Phenolic Acids from *Artemisia argyi* Leaves by Tailor-Made Ternary Deep Eutectic Solvents

**DOI:** 10.3390/molecules24152842

**Published:** 2019-08-05

**Authors:** Li Duan, Chenmeng Zhang, Chenjing Zhang, Zijing Xue, Yuguang Zheng, Long Guo

**Affiliations:** 1 College of Chemistry and Material Science, Hebei Normal University, Shijiazhuang 050024, China; 2 School of Pharmacy, Hebei University of Chinese Medicine, Shijiazhuang 050200, China

**Keywords:** deep eutectic solvents, *Artemisia argyi* leaves, phenolic acids, extraction, response surface methodology

## Abstract

The *Artemisia argyi* leaf (AL) has been used as a traditional medicine and food supplement in China and other Asian countries for hundreds of years. Phytochemical studies disclosed that AL contains various bioactive constituents. Among bioactive constituents, phenolic acids have been recognized as the main active compounds in AL. To the best of our knowledge, no research has been focused on extraction method for the bioactive phenolic acids from AL. Nowadays, deep eutectic solvents (DESs) are emerging as a new type of green and sustainable solvent for efficient extraction of bioactive compounds from natural products. In the present study, an environmentally friendly extraction method based on DESs was established to extract bioactive phenolic acids from ALs. Diverse tailor-made solvents, including binary and ternary DESs, were explored for simultaneous extraction of four phenolic acids (3-caffeoylquinic acid, 3,4-di-*O*-caffeoylquinic acid, 3,5-di-*O*-caffeoylquinic acid, and 4,5-di-*O*-caffeoylquinic acid) from AL. The results indicated that the ternary DES composed of a 2:1:2 molar ratio of choline chloride, malic acid, and urea showed enhanced extraction yields for phenolic acids compared with conventional organic solvents and other DESs. Subsequently, the extraction parameters for the four phenolic acids by selected tailor-made DESs, including liquid–solid ratios, water content (%) in the DESs, and extraction time, were optimized using response surface methodology and the optimal extraction conditions were: extraction time, 23.5 min; liquid–solid ratio, 57.5 mL/g (mL of DES/g dry weight of plant material); water content, 54%. The research indicated that DESs were efficient and sustainable green extraction solvents for extraction of bioactive phenolic acids from natural products. Compared to the conventional organic solvents, the DESs have a great potential as possible alternatives to those organic solvents in health-related areas such as food and pharmaceuticals.

## 1. Introduction

The *Artemisia argyi* leaf (AL), which is widely distributed in China and other Asian countries, has been used as a traditional medicine or food supplement for hundreds of years [1]. As a traditional Chinese medicine, AL is reported to possess antioxidant, antibacterial, anti-inflammatory, anticancer, hemostatic, and analgesic activities and is commonly used for treatment of hemorrhage, pain, eczema, and menstruation-related symptoms [2,3,4]. AL is also consumed as a food ingredient because of its delicious flavor and distinctive smell. In China, AL is used as a common condiment and colorant for the traditional Chinese food “Qingtuan”. In Japan, AL is added into food as an additive to enhance the flavor and nutrition [5]. 

Although the AL has been used as an herbal medicine and food ingredient for a long time, studies on its bioactive compositions are still limited. Phytochemical studies disclosed that AL contains various bioactive constituents, mainly including volatile oils, phenolic acids, flavonoids, and terpenoids [6,7]. Among bioactive constituents, phenolic acids have been recognized as the main active compounds in AL [8,9]. Therefore, the content of the phenolic acids is an important index for quality analysis and normal applications of AL. To date, several bioactive phenolic acids, such as 3-caffeoylquinic acid, 3,4-di-*O*-caffeoylquinic acid, 3,5-di-*O*-caffeoylquinic acid, and 4,5-di-*O*-caffeoylquinic acid, have been isolated from AL. A number of analytical methods, including high-performance liquid chromatography (HPLC) and high-performance liquid chromatography coupled with mass spectrometry (HPLC-MS), have been used for qualitative and quantitative analysis of the main phenolic acids in AL [5,7]. However, the research focused on extraction methods for bioactive phenolic acids from AL is still limited.

Nowadays, conventional organic solvents, such as alcohols, ethyl acetate, acetone, and chloroform, are widely used in the extraction of bioactive components from natural sources [10]. However, the consumption of large amounts of these volatile and hazardous organic solvents may contribute to environmental pollution and leave unacceptable solvent residues in extracts. Therefore, in analytical chemistry, green extraction methods which are environmentally friendly and sustainable for sample preparation have received more and more attention [11]. Since being introduced as a new type of green solvent, deep eutectic solvents (DESs) have rapidly gained great interest as sustainable alternatives to conventional organic solvents. DESs are prepared by simply mixing two or more naturally occurring, inexpensive, and biodegradable components together to obtain a eutectic mixture [12]. The availability, low cost, biodegradability, and environmental friendliness of the components make the DESs versatile alternatives to conventional organic solvents [13]. Due to their excellent properties, including biodegradability, low toxicity, solute stabilization, and low cost, DESs have been widely used in organic synthesis, separation processes, and biomedical applications [14,15]. 

Generally, DESs are prepared by simply mixing two or more naturally occurring, inexpensive, and biodegradable components together to obtain eutectic mixtures. As tunable solvents, diverse possible combinations of starting components have different targeted functionality, which means that we can increase the solubility and extraction efficiency of DESs for target compounds by selecting appropriate combinations of starting components. The tailor-made DESs have tremendous potential for efficient and simultaneous extraction and separation of compounds which have obvious differences in nature [16]. Recently, many reports have shown that tailor-made DESs were successfully employed in the extraction and separation of different kinds of bioactive compounds, such as phenolic acids, flavonoids, alkaloids, and saponins, from various plant materials [17,18,19,20,21,22]. Nonetheless, most of the research used tailor-made binary DESs as extraction solvents for bioactive compound extractions, the number of reports on the application of tailor-made ternary DESs for extraction is still limited, and the efficiency of DESs for extraction of bioactive phenolic acids from AL still remains unknown.

In the present study, in order to evaluate DESs for the extraction of phenolic acids, several tailor-made binary and ternary DESs were used for simultaneous extraction of four bioactive phenolic acids (3-caffeoylquinic acid, 3,4-di-*O*-caffeoylquinic acid, 3,5-di-*O*-caffeoylquinic acid, and 4,5-di-*O*-caffeoylquinic acid) from AL, and the extraction efficiency of tailor-made DESs was compared with that of conventional organic solvents. Moreover, the extraction parameters for phenolic acids by tailor-made DESs were systematically optimized using response surface methodology (RSM).

## 2. Results and Discussion

### 2.1. Chromatographic Conditions and Method Validation

**Figure 1:**
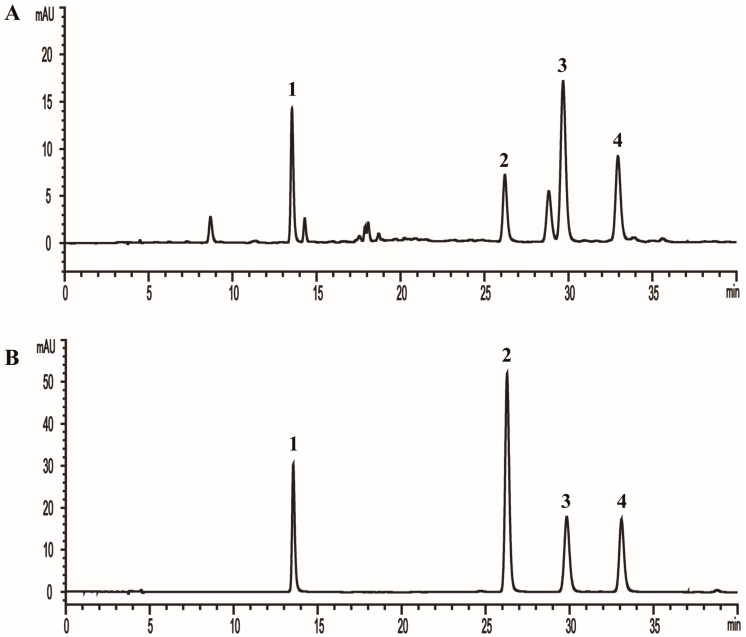
The typical HPLC chromatograms of (**A**) *Artemisia argyi* leaves sample (20 mg/mL) and (**B**) four phenolic acids reference standards. (1. 3-caffeoylquinic acid, 4.96 μg/mL; 2. 3,4-di-*O*-caffeoylquinic acid, 4.24 μg/mL; 3. 3,5-di-*O*-caffeoylquinic acid, 5.36 μg/mL; 4. 4,5-di-*O*-caffeoylquinic acid, 4.16 μg/mL).

In order to achieve a rapid and efficient analysis of the four phenolic acids (3-caffeoylquinic acid, 3,4-di-*O*-caffeoylquinic acid, 3,5-di-*O*-caffeoylquinic acid, and 4,5-di-*O*-caffeoylquinic acid) in AL, different mobile phases (including water–methanol, water–acetonitrile, formic acid water–methanol, and formic acid water–acetonitrile), flow rates (0.7 mL/min, 0.8 mL/min, and 1.0 mL/min), as well as column temperatures (15 °C, 20 °C, 25 °C, and 30 °C, Supplementary Materials Figure S1) were examined and compared. As a result, the formic acid water–acetonitrile system at 15 °C with a flow rate of 0.7 mL/min was finally selected for the suitable analysis duration, greater separation ability, and better peak shapes. The gradient elution was as follows: 0–5 min, 12% B; 5–15 min, 12–22% B; 15–25 min, 22% B; 25–35 min, 22–25% B; 35–40 min, 25–40% B. The typical HPLC chromatograms of the AL sample and four phenolic acids reference standards are shown in Figure 1.

Method validation of quantitative analysis was performed. The linearity, limit of detections (LODs), limit of quantifications (LOQs), precision, repeatability, stability, and accuracy for the four phenolic acids were validated. Each calibration curve was performed with six different concentrations in triplicate. All calibration curves were of good linearity with high correlation coefficient (R^2^ > 0.9997) over the tested range. The LODs and LOQs of the four analytes were defined by the concentration that generated peaks with signal-to-noise values of 3 and 10 using standard solutions. The precision of the developed method was determined by the intra- and interday variations. For the intraday test, the sample was analyzed six times within the same day, while for the interday test, the sample was examined in duplicates for three consecutive days. The relative standard deviations (RSDs) of intraday and interday precisions were less than 1.61% and 2.14%, respectively. For the repeatability test, six replicates of the same sample were prepared and analyzed, and for the stability test, the same sample was stored at room temperature and analyzed by replicate injection analysis at 0, 2, 4, 8, 12, and 24 h. The repeatability presented as RSDs was less than 2.71%, and the stability was less than 2.06%. The recovery was used to evaluate the accuracy of the method. Known amounts of the four phenolic acids standard solutions were added into the same samples in sextuplicate, and then extracted and analyzed with the same procedures. The recovery of each analyte was calculated by the equation: Recovery (%) = (Detected amount − Original amount)/Spiked amount × 100%. The overall recoveries of the four analytes were in the range of 101.15–102.86% with RSDs less than 1.67%. The data of method validation are shown in Supplementary Materials Table S1. 

### 2.2. Screening of DESs for the Extraction of Phenolic Acids from AL

#### 2.2.1. Extraction of Phenolic Acids by Binary DESs

DESs are composed of a mixture consisting of hydrogen bond acceptors (HBAs) with hydrogen bond donors (HBDs). The composition of DESs determines their physicochemical properties and consequently greatly influences extraction efficiency of natural compounds [23]. In the present study, six choline-chloride-based binary DESs, ChCl-Ma, ChCl-Ur, ChCl-Ga, ChCl-Pa, ChCl-Eg, and ChCl-Gl, were successfully synthesized (Table 1) and selected to test their extraction efficiency for phenolic acids. The high viscosity of most DESs at room temperature restricted their application due to a slow mass transfer. To overcome this problem, extraction conditions were adjusted to reduce the viscosity by increasing extraction temperature and adding a certain amount of water [24]. In the initial screening experiments, 75% DES solution in water (*v*/*v*) was employed, and the extraction conditions were as follows: extraction time, 30 min; extraction temperature, 50 °C; liquid–solid ratio, 50 mL/g (mL of DES/g dry weight of plant material). 

**Figure 2:**
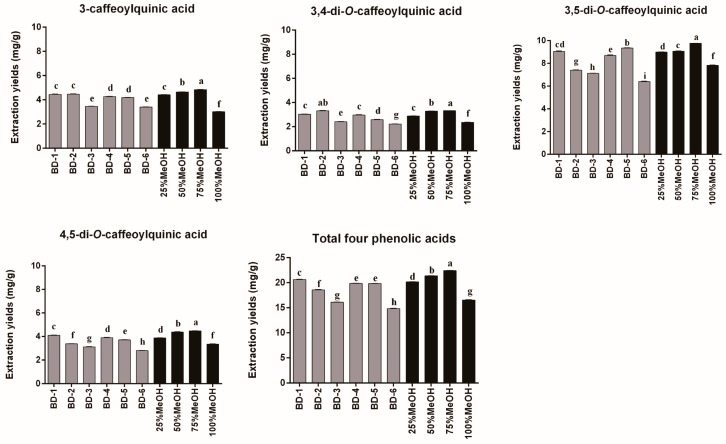
Extraction yields of different binary DESs and different ratios of methanol for 3-caffeoylquinic acid, 3,4-di-*O*-caffeoylquinic acid, 3,5-di-*O*-caffeoylquinic acid, 4,5-di-*O*-caffeoylquinic acid, and total four phenolic acids from *Artemisia argyi* leaves (*n* = 3). Numbers on horizontal axis are in accordance with the numbers in Table 1. Error bars indicate the SD (*n* = 3). Extraction yields which do not share the same letter are significantly different (*p* < 0.05).

Extraction yields of the four phenolic acids (3-caffeoylquinic acid, 3,4-di-*O*-caffeoylquinic acid, 3,5-di-*O*-caffeoylquinic acid, and 4,5-di-*O*-caffeoylquinic acid) with different binary DESs are shown in Figure 2. The initial screening results indicated that the extraction efficiency for phenolic acids was influenced by the types of DES solvents, and different types of DESs resulted in different extraction yields. In general, extraction yields of the four phenolic acids followed the order 3,5-di-*O*-caffeoylquinic acid > 3-caffeoylquinic acid > 4,5-di-*O*-caffeoylquinic acid > 3,4-di-*O*-caffeoylquinic acid. The phenolic acid, 3,5-di-*O*-caffeoylquinic acid, showed high extraction efficiency in ChCl-Eg with the concentration 9.35 ± 0.03 mg/g (mg/g dry weight of plant material), followed by ChCl-Ma (9.05 ± 0.05 mg/g) and ChCl-Pa (8.71 ± 0.03 mg/g). For 3-caffeoylquinic acid, ChCl-Ma (4.45 ± 0.03 mg/g) and ChCl-Ur (4.47 ± 0.03 mg/g) led to higher extraction yields. For 4,5-di-*O*-caffeoylquinic acid, ChCl-Ma (4.10 ± 0.02 mg/g), ChCl-Pa (3.90 ± 0.03 mg/g), and ChCl-Eg (3.72 ± 0.02 mg/g) exhibited higher extraction efficiency. For 3,4-di-*O*-caffeoylquinic acid, ChCl-Ur (3.32 ± 0.03 mg/g) showed higher extraction efficiency, followed by ChCl-Ma (3.03 ± 0.02 mg/g) and ChCl-Pa (2.97 ± 0.03 mg/g).In conclusion, the extraction yields of the total phenolic acids were calculated, and it was clearly shown that ChCl-Ma (BD-1) was the best binary DES for extraction of four phenolic acids from AL with the extraction yields 20.64 ± 0.08 mg/g.

In order to comprehensively compare the extraction efficiency of DESs and conventional solvents for extraction of phenolic acids from AL, different ratios of methanol (25% MeOH, 50% MeOH, 75% MeOH, and 100% MeOH), efficient solvents commonly used in the extraction of bioactive compounds from natural products, were selected as reference solvents [7]. As shown in Figure 2, among the different ratios of MeOH, 75% MeOH displayed the highest extraction efficiency for the four phenolic acids. It was clear that all the six tailor-made binary DESs exhibited higher extraction efficiency for the four phenolic acids compared with 100% MeOH (16.56 ± 0.06 mg/g). However, compared to 75% MeOH (22.41 ± 0.03 mg/g), none of the six binary DESs exhibited a higher extraction yield. Based on the results above, ChCl-Ma (BD-1) was selected as the best binary DES for extraction of phenolic acids from AL, and we attempted to synthesize a series of ternary DESs based on ChCl-Ma to enhance extraction efficiency of phenolic acids in further tests.

#### 2.2.2. Extraction of Phenolic Acids by Ternary DESs

**Figure 3:**
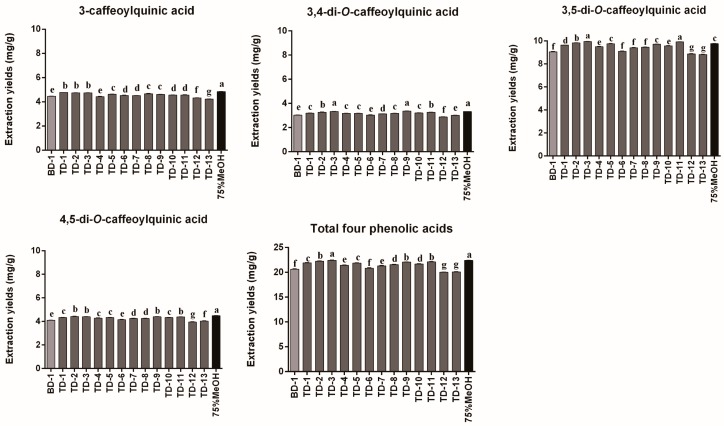
Extraction yields of different ternary DESs for 3-caffeoylquinic acid, 3,4-di-*O*-caffeoylquinic acid, 3,5-di-*O*-caffeoylquinic acid, 4,5-di-*O*-caffeoylquinic acid, and total four phenolic acids from *Artemisia argyi* leaves (*n* = 3). Numbers on horizontal axis are in accordance with the numbers in Table 1 and Table 2. Error bars indicate the SD (*n* = 3). Extraction yields which do not share the same letter are significantly different (*p* < 0.05).

DESs can be synthesized from two or more components. According to the previous report, the ternary DESs forming with addition of glycerol to the binary DESs show lower melting points and viscosities [25]. Adding the third component to binary DESs may change the properties of ternary DESs and thus influence the extraction yields. In this study, based on the best binary DES (ChCl-Ma) selected above, several tailor-made ternary DESs were designed in order to further enhance the extraction yields of phenolic acids from AL. Five ternary DESs, including ChCl-Ma-Ur, ChCl-Ma-Ga, ChCl-Ma-Pa, ChCl-Ma-Eg, and ChCl-Ma-Gl, were successfully synthesized (Table 2) and further used to test their extraction efficiency for phenolic acids. The extraction yields of the four phenolic acids employing the tailor-made ternary DESs are listed in Figure 3. The results indicated that compared to the binary DESs, some tailor-made ternary DESs could change the extraction efficiency. Almost all of the tailor-made ternary DESs exhibited higher extraction efficiency for the four phenolic acids compared with the best binary DES (ChCl-Ma), except ChCl-Ma-Gl (TD-12 and TD-13). It could be speculated that the addition of the third component might reduce the viscosity of DESs, enhancing the hydrogen bond interactions between DESs and the target components, thus improving the extraction yields [26]. 

Several studies have revealed that different molar ratios of DES components result in different viscosities and surface tensions, thereby affecting the extraction efficiency of target components from natural biomass [27]. Thus, in the study, the effects of molar ratios of tailor-made ternary DESs were also investigated. As shown in Figure 3, the optimal molar ratio of ChCl-Ma-Ur was 2:1:2 (TD-3) and the extraction yields of the four phenolic acids were 22.43 ± 0.02 mg/g. The optimal molar ratio of ChCl-Ma-Ga was 2:2:1 (TD-5) and the extraction yields of the four phenolic acids were 21.89 ± 0.02 mg/g. The optimal molar ratio of ChCl-Ma-Pa was 2:1:2 (TD-9) and the extraction yields of the four phenolic acids were 22.08 ± 0.03 mg/g. The optimal molar ratio of ChCl-Ma-Eg was 2:2:1 (TD-11) and the extraction yields of the four phenolic acids were 22.12 ± 0.05 mg/g. The optimal molar ratio of ChCl-Ma-Gl was 2:2:1 (TD-13) and the extraction yields of the four phenolic acids were 20.09 ± 0.04 mg/g. None of the tailor-made ternary DESs with different molar ratios exhibited an extraction yield higher than 75% MeOH, the best conventional solvent. However, the tailor-made ternary DES, ChCl-Ma-Ur (2:1:2), produced similar extraction efficiency for the four phenolic acids compared to 75% MeOH (*p* > 0.05). Based on the results, the ChCl-Ma-Ur with a molar ratio of 2:1:2 (TD-3) was selected as the best ternary DES for extraction of phenolic acids from AL.

### 2.3. Optimization of the Extraction Parameters for Phenolic Acids

The above extraction investigations showcased that the tailor-made ternary DES, ChCl-Ma-Ur (2:1:2), was selected as the best ternary DES for extraction of phenolic acids from AL. In order to obtain the optimal extraction efficiency for phenolic acids from AL, several numerical variables that could affect the extraction efficiencies were optimized by RSM, a valuable statistical technique to determine the optimal values of the independent variables and to enable the user to effectively investigate the effects of multiple factors [28,29]. Similar to previous studies [30], three variables of extraction time, liquid–solid ratios, and water content in DESs were evaluated using Box–Behnken design (BBD). After determining the range of extraction factors on the basis of preliminary single-factor test, the extraction time (A), liquid–solid ratios (B), and water content (C) were varied at three levels (−1, 0, +1) as follows: A, 8.0–40.0 min; B, 17.5–57.5 mL/g; C 20–70%. The total extraction amounts of the four phenolic acids were taken as the responses of the design experiments. The experimental orders, levels of variables, and response values are summarized in Table 3. 

Experiments conducted according to the design resulted in a second-order polynomial equation for total extraction amounts (Y) expressed using coded variables (A, B, and C) as follows:

Y = 22.16 − 0.20A + 0.095B + 1.56C − 0.11AB − 0.27AC + 1.24BC − 0.14A^2^ + 0.027B^2^ − 3.76C^2^

The model was evaluated in terms of the square of correlation coefficient (R^2^) and the lack of fit by the analysis of variance (ANOVA) at the 95% confidence level (Table 4). The resulting R^2^ value was 0.9765, indicating that the experimental data were in relatively good agreement with predicted extraction yields. The lack-of-fit value, which evaluates the failure of the model to represent the data in the experimental domain points, was insignificant for the response with *p*-value of 0.3789 (*p* > 0.05).

**Figure 4:**
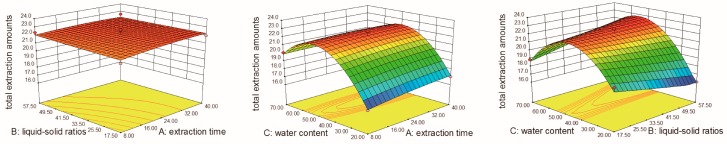
Response surface plots of the model for extraction of phenolic acids from *Artemisia argyi* leaves. (A. extraction time, min; B. liquid–solid ratios, mL/g; C. water content, %).

Statistical analysis and 3D response plots (Figure 4) illustrated the significant variables affecting extraction yields of phenolic acids and the interaction effects between the variables. In the model, the water content (C) showed significant effects on the extraction efficiency of phenolic acids (*p* < 0.0001). Based on the adequate model, the calculated optimum conditions for the extraction of phenolic acids from AL were as follows: extraction time, 23.5 min; liquid–solid ratio, 57.5 mL/g; water content, 54%. Triplicate experiments were carried out under the optimal extraction conditions and mean values of experimental results were compared with the predicted values. Under the optimum conditions, the total extraction amounts of the four phenolic acids were 22.80 mg/g, which were closed to the predicted values of 22.79 mg/g. The results obtained through confirmation experiments indicated the model is adequate for predicting the expected optimization.

## 3. Materials and Methods 

### 3.1. Materials and Reagents

AL samples were purchased from a Chinese herbal medicine market (Anguo, China). The samples were dried in the shade and stored in the desiccator. The AL samples were authenticated by Prof. Yuguang Zheng from Department of Pharmacognosy, Hebei University of Chinese Medicine and the voucher specimens were deposited in Hebei University of Chinese Medicine, Shijiazhuang, China.

**Figure 5:**
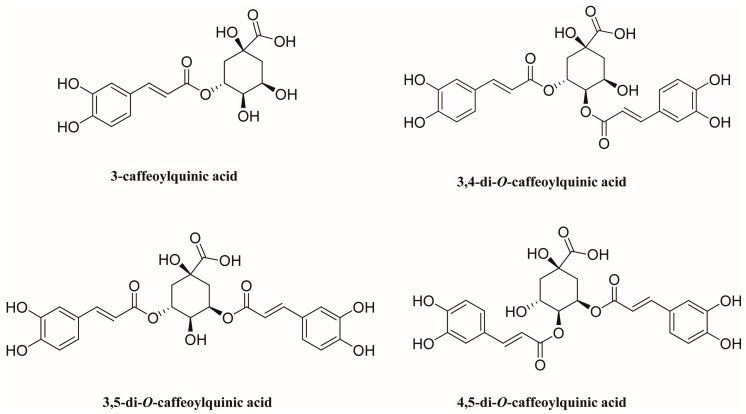
Chemical structures of 3-caffeoylquinic acid, 3,4-di-*O*-caffeoylquinic acid, 3,5-di-*O*-caffeoylquinic acid, and 4,5-di-*O*-caffeoylquinic acid.

Four phenolic acids reference compounds, 3-caffeoylquinic acid, 3,4-di-*O*-caffeoylquinic acid, 3,5-di-*O*-caffeoylquinic acid, and 4,5-di-*O*-caffeoylquinic acid, were purchased from Chengdu Must Bio-technology Co., Ltd. (Chengdu, China). The purities of the four reference compounds were determined to be higher than 98% by high-performance liquid chromatography diode array detection analysis. The structures of the four phenolic acids are shown in Figure 5.

Chemical compounds for DES preparation including choline chloride (ChCl), DL-malic acid (Ma), urea (Ur), glutaric acid (Ga), propanedioic acid (Pa), ethylene glycol (Eg), glycerol (Gl) were obtained from Aladdin Reagent Company (Shanghai, China). Acetonitrile, methanol, and formic acid (chromatographic grade) were purchased from Merck (Darmstadt, Germany). Deionized water was prepared by a Milli-Q water purification system (Millipore, Billerica, MA, USA). Other reagents and chemicals used in this work were of analytical grade. 

### 3.2. HPLC Analysis

HPLC analysis was performed on an Agilent 1260 HPLC system equipped with a quaternary pump, a degasser, an autosampler, a thermostated column compartment, and a diode array detector (Agilent Technologies, Palo Alto, CA, USA). Chromatographic separation was achieved on an Agilent SB C18 column (4.6 × 250 mm, 5 μm). The mobile phase was composed of 0.1% formic acid water (A) and acetonitrile (B) with a gradient elution as follows: 0–5 min, 12% B; 5–15 min, 12–22% B; 15–25 min, 22% B; 25–35 min, 22–25% B; 35–40 min, 25–40% B. The detection wavelength was 330 nm. The flow rate was set at 0.7 mL/min and the column temperature was set at 15 °C. 

### 3.3. Preparation of DESs

The DESs were synthesized according to the previous studies [31]. Briefly, different DESs were obtained by simply mixing hydrogen bond acceptors (HBAs) and hydrogen bond donors (HBDs) together in a proper molar ratio with constant stirring at 80 °C until a clear and homogeneous liquid formed. 

### 3.4. Extraction of Phenolic Acids From AL

The AL samples were powdered and screened through 40 mesh sieves. An accurately weighed powder (20 mg) was extracted with 1 mL of different solvents (different DESs, 25% MeOH, 50% MeOH, 75% MeOH, and 100% MeOH) in a 2 mL centrifuge tube by ultrasonic cleaner (KQ5200B, 200W, 40 kHz, Kunshan, China) for 30 min. After extraction, the extracted solution was centrifuged at 13000 rpm/min for 10 min. Then, 0.1 mL of supernatant was sampled and diluted 10-fold with methanol. The diluted solution (2 μL) was injected into the HPLC instrument for analysis. Three replicates of each sample were prepared and analyzed (*n* = 3).

### 3.5. DESs Tailoring

In order to determine the effect of the DES compositions on the extraction of the four phenolic acids from AL, six ChCl-based binary DESs, including ChCl-Ma, ChCl-Ur, ChCl-Ga, ChCl-Pa, ChCl-Eg, and ChCl-Gl, were successfully synthesized in initial screening. Then, the extraction efficiency of the different binary DESs for the four phenolic acids was investigated and the optimal binary DES was selected. Next, different efficient components were added into the optimal binary DES obtained in initial screening to design different ternary DESs. Five tailored ternary DESs with different molar ratios, including ChCl-Ma-Ur, ChCl-Ma-Ga, ChCl-Ma-Pa, ChCl-Ma-Eg, and ChCl-Ma-Gl, were synthesized and the extraction efficiencies for the four phenolic acid compounds were tested.

### 3.6. Optimization of the DES Extraction Parameters for Phenolic Acids 

The optimal extraction parameters for the phenolic acids from AL were obtained using RSM. RSM was performed using the Design-Expert Ver. 8.0.6 (Stat-Ease Inc., Minneapolis, MN, USA). After determining the range of extraction variables on the basis of preliminary single-factor test, Box–Behnken design (BBD) was used to find the optimal values for three independent variables: extraction time (A), liquid–solid ratios (B), and water content (%) in DESs (C). The total extraction amounts of the four phenolic acids were taken as the response of the design experiments. Regression analysis was performed according to the experimental data. Subsequently, additional confirmation experiments were conducted to confirm the validity of the statistical experimental strategies.

## 4. Conclusions

In this work, a green and efficient extraction method using tailor-made DESs as extraction solvents was established for extraction of bioactive phenolic acids from AL. Six binary DESs and five ternary DESs were successfully synthesized and used to extract four phenolic acids from AL. The results indicated that the tailor-made DESs were efficient solvents for the extraction of phenolic acids. The ternary DES, ChCl-Ma-Ur with a molar ratio of 2:1:2, proved to be the most efficient solvent for phenolic acid extraction from AL. Moreover, the optimal extraction conditions for phenolic acids by ChCl-Ma-Ur (2:1:2) were determined using RSM and the optimal extraction conditions are extraction time, 23.5 min; liquid–solid ratio, 57.5 mL/g; and water content, 54%. The present study suggests that the DES-based extraction method is efficient and sustainable for extraction of bioactive phenolic acids from AL. The DESs are truly designed and efficient solvents which could be used as green solvents for the extraction of bioactive compounds from natural products and have a great potential as possible alternatives to those organic solvents in health-related areas such as food and pharmaceuticals.

## Figures and Tables

**Table 1:** The binary DESs synthesized in this study.

NO.	Abbreviation	Component 1	Component 2	Molar Ratio
BD-1	ChCl-Ma	choline chloride	dl-malic acid	1:1
BD-2	ChCl-Ur	choline chloride	urea	1:2
BD-3	ChCl-Ga	choline chloride	glutaric acid	1:1
BD-4	ChCl-Pa	choline chloride	propanedioic acid	1:1
BD-5	ChCl-Eg	choline chloride	ethylene glycol	1:3
BD-6	ChCl-Gl	choline chloride	glycerol	1:2

**Table 2:** The ternary DESs synthesized in this study.

NO.	Abbreviation	Component 1	Component 2	Component 3	Molar Ratio	Extraction Yield of Four Phenolic Acids (mg/g)
TD-1	ChCl-Ma-Ur	choline chloride	DL-malic acid	urea	2:1:1	21.92 ± 0.04
TD-2	ChCl-Ma-Ur	choline chloride	DL-malic acid	urea	2:2:1	22.26 ± 0.04
TD-3	ChCl-Ma-Ur	choline chloride	DL-malic acid	urea	2:1:2	22.43 ± 0.02
TD-4	ChCl-Ma-Ga	choline chloride	DL-malic acid	glutaric acid	2:1:1	21.41 ± 0.04
TD-5	ChCl-Ma-Ga	choline chloride	DL-malic acid	glutaric acid	2:2:1	21.89 ± 0.02
TD-6	ChCl-Ma-Pa	choline chloride	DL-malic acid	propanedioic acid	2:2:1	20.81 ± 0.06
TD-7	ChCl-Ma-Pa	choline chloride	DL-malic acid	propanedioic acid	2:1:1	21.30 ± 0.05
TD-8	ChCl-Ma-Pa	choline chloride	DL-malic acid	propanedioic acid	1:1:1	21.55 ± 0.04
TD-9	ChCl-Ma-Pa	choline chloride	DL-malic acid	propanedioic acid	2:1:2	22.08 ± 0.03
TD-10	ChCl-Ma-Eg	choline chloride	DL-malic acid	ethylene glycol	1:2:0.5	21.67 ± 0.04
TD-11	ChCl-Ma-Eg	choline chloride	DL-malic acid	ethylene glycol	2:2:1	22.12 ± 0.05
TD-12	ChCl-Ma-Gl	choline chloride	DL-malic acid	glycerol	1:2:0.5	20.02 ± 0.02
TD-13	ChCl-Ma-Gl	choline chloride	DL-malic acid	glycerol	2:2:1	20.09 ± 0.04

**Table 3:** The experimental orders, levels of variables, and response values in Box–Behnken design.

Run	Factors			Responses
	Extraction Time (A, min)	Liquid–Solid Ratios (B, mL/g)	Water Content (C, %)	Total Extraction Amounts of Four Phenolic Acids (mg/g)
1	24.0	37.5	45	22.60
2	40.0	57.5	45	22.61
3	24.0	17.5	20	18.47
4	8.0	37.5	20	16.13
5	24.0	37.5	45	22.10
6	40.0	17.5	45	21.96
7	24.0	57.5	70	20.85
8	24.0	37.5	45	21.40
9	8.0	57.5	45	22.34
10	24.0	17.5	70	18.86
11	24.0	57.5	20	15.51
12	24.0	37.5	45	22.65
13	8.0	17.5	45	21.27
14	40.0	37.5	70	19.84
15	24.0	37.5	45	22.02
16	8.0	37.5	70	20.06
17	40.0	37.5	20	17.00

**Table 4:** The ANOVA results of the quadratic multiple regression model for phenolic acids.

Source	Sum of Squares	df	Mean Square	F Value	*p*-Value Prob > F	Significance
Model	86.54	9	9.62	32.38	<0.0001	significant
A	0.32	1	0.32	1.08	0.3323	
B	0.073	1	0.073	0.24	0.6359	
C	19.53	1	19.53	65.78	<0.0001	
AB	0.045	1	0.045	0.15	0.7085	
AC	0.3	1	0.3	1.02	0.3468	
BC	6.13	1	6.13	20.64	0.0027	
A^2^	0.08	1	0.08	0.27	0.6188	
B^2^	3.10 × 10^−3^	1	3.10 × 10^−3^	0.01	0.9215	
C^2^	59.51	1	59.51	200.42	<0.0001	
Residual	2.08	7	0.3			
Lack of Fit	1.04	3	0.35	1.34	0.3789	not significant
Pure Error	1.04	4	0.26			
R^2^	0.9765					

## Supplementary Materials

The Supplementary Materials are available online.
