# Supplementary material for: Green Extraction of Phenolic Acids from Artemisia argyi Leaves by Tailor-Made Ternary Deep Eutectic Solvents
Source: Molecules. 2019 Aug 5;24(15):2842. doi: 10.3390/molecules24152842 (PMC6695999; doi:10.3390/molecules24152842)
Supplement: Supplementary file 1 [file molecules-24-02842-s001.pdf]

Article

Green Extraction of Phenolic Acids from *Artemisia argyi* Leaves by Tailor-Made Ternary Deep Eutectic Solvents

Li Duan [1,†], Chenmeng Zhang [1,†], Chenjing Zhang [1], Zijing Xue [2], Yuguang Zheng [2,*] and Long Guo [2,*]

[1] College of Chemistry and Material Science, Hebei Normal University, Shijiazhuang, Hebei 050024, China

[2] School of Pharmacy, Hebei University of Chinese Medicine, Shijiazhuang, Hebei 050200, China

* Correspondence: zyg314@163.com (Y.Z.); guo_long11@163.com (L.G.); Tel.: +86-0311-89926316 (Y.Z.); +86-0311-89926316 (Y.Z.); +86-0311-89926017 (L.G.)

† These authors contributed equally to this work.

Table S1. The linearity, limit of detections (LODs), limit of quantifications (LOQs), precision, stability and accuracy of the four phenolic acids.

NO.	Analytes	Calibration curve	R^2	Linear range (μg/mL)	LODs (μg/mL)	LOQs (μg/mL)	Precision (RSDs, %)		Repeatability (*n* = 6)	Stability (*n* = 6, 24 h)	Recovery (n = 6)	
							Intra-Day (n=6)	Inter-day (n=9)	RSDs, %	RSDs, %	Recovery, %	RSD, %
1	3-caffeoylquinic acid	y = 8.2847x - 1.0192	0.9999	1.40-112	0.66	1.40	1.52	2.11	2.54	1.22	101.90	1.67
2	3,4-di-*O*-caffeoylquinic acid	y = 9.201x - 2.3121	0.9999	1.16-93	0.46	1.16	1.61	1.83	2.71	1.39	102.86	0.81
3	3,5-di-*O*-caffeoylquinic acid	y = 10.074x - 3.7888	0.9999	1.42-114	0.68	1.42	1.45	2.06	1.77	2.06	101.15	1.42
4	4,5-di-*O*-caffeoylquinic acid	y = 11.054x - 7.3871	0.9997	1.15-94	0.42	1.15	1.23	2.14	2.41	1.80	102.60	1.03

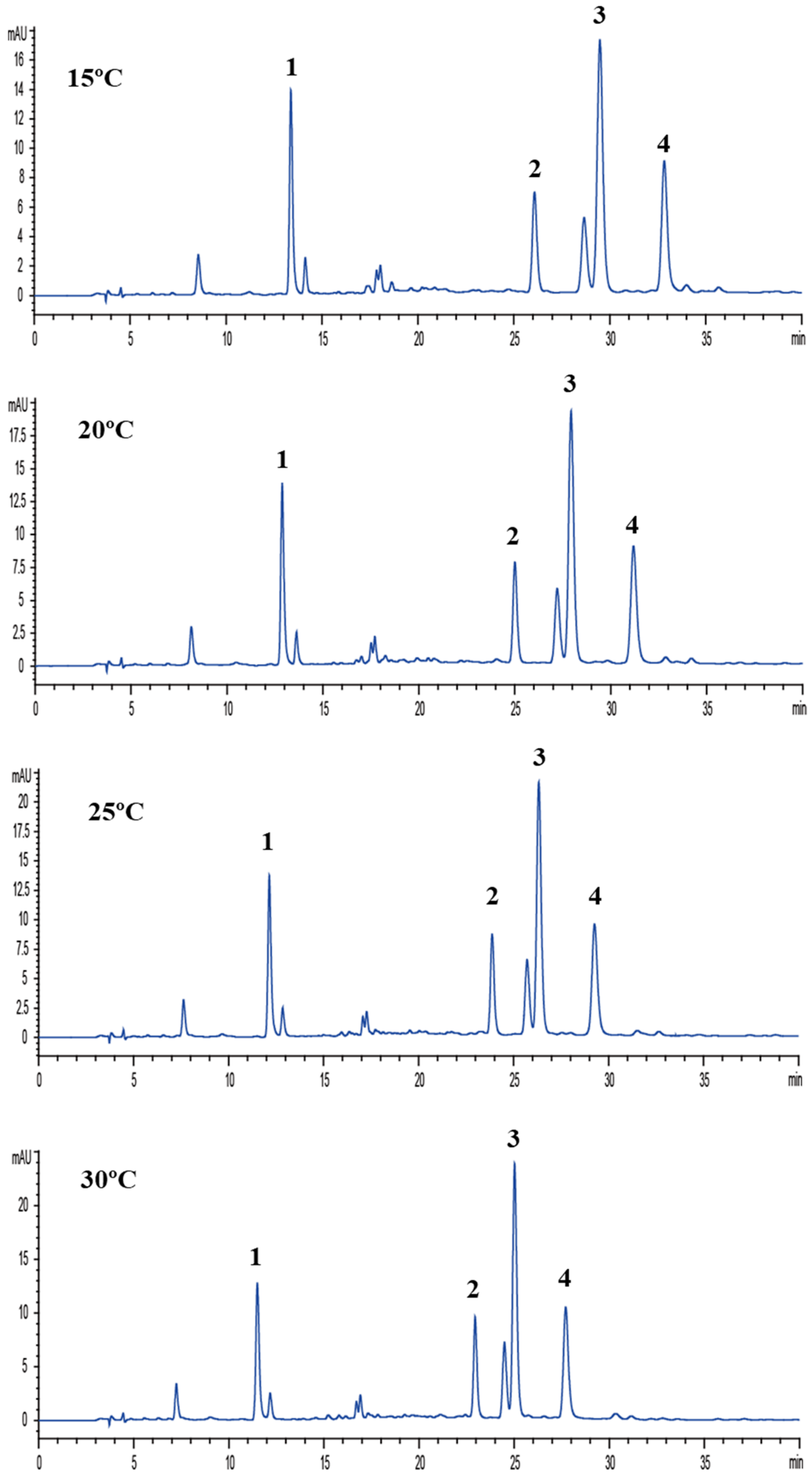

Figure S1. The HPLC chromatograms of *Artemisia argyi* leaves sample at different column temperatures. (1. 3-caffeoylquinic acid, 2. 3,4-di-O-caffeoylquinic acid, 3. 3,5-di-O-caffeoylquinic acid, 4. 4,5-di-O-caffeoylquinic acid)
